# eHealth Literacy and Trust in Health Information Sources

**DOI:** 10.3390/healthcare13060616

**Published:** 2025-03-12

**Authors:** Abdullah Alhewiti

**Affiliations:** Department of Family and Community Medicine, Faculty of Medicine, University of Tabuk, Tabuk 71491, Saudi Arabia; aalhewiti@ut.edu.sa

**Keywords:** eHealth literacy, internet, health information, healthcare workers, healthcare providers, trust

## Abstract

Introduction: The spread of health-related information across the internet necessitates an evaluation of public eHealth literacy, trust in different health information sources, including healthcare providers, and how eHealth literacy is related to trust in different sources. Methods: 407 individuals participated in a web-based survey in the Tabuk region of Saudi Arabia. Univariate analysis was used to evaluate the relationships between eHealth literacy and demographic variables, and multiple linear regression was used to measure the relationship between eHealth literacy and trust in health information sources after adjustment for demographic factors. Results: The average eHealth literacy of the respondents was 27.17 out of 40. eHealth literacy levels were higher among females, younger age groups, those in the higher-education category, and those with a chronic disease or currently on medication. For 51.9% of participants, physicians and healthcare workers were their main source of health information, while 40% considered the internet their main source. None of the study participants perceived physicians and healthcare workers as untrustworthy, and social media was the least trusted source. eHealth literacy was not related to trust in physicians and health workers but was positively associated with trust in specialized health websites and negatively associated with trust in social media. Conclusions: The findings suggest that the public tends to prefer and trust physicians and other healthcare workers as a primary source of health information, regardless of their eHealth literacy levels. A higher eHealth literacy level was associated with trust in specialized health websites and distrust in social media.

## 1. Introduction

Health information plays a fundamental role in shaping public health behaviors by influencing individuals’ beliefs, attitudes, and choices related to their health. The dissemination of accessible, accurate, and timely health information empowers individuals to make informed decisions, embrace a healthier lifestyle, engage in their own healthcare, and implement preventive measures. The internet’s rapid expansion and widespread adoption have significantly influenced the communication and dissemination of health information. As online health resources become increasingly accessible, a growing number of individuals are turning to digital platforms to obtain health information [1]. This has inevitably been seen in Saudi Arabia, where 99% of the population has access to the internet, and individuals from different backgrounds are receiving health advice from the internet because of the convenience and ease of access [2,3].

“eHealth” (electronic health) literacy refers to a person’s ability to locate, comprehend, analyze, and utilize health-related information from electronic sources and apply the acquired knowledge to address or resolve health-related issues [4]. Before the COVID-19 pandemic, eHealth literacy existed as an emerging but underprioritized competency in an era of slow and gradual digital healthcare integration [5]. The advent of the pandemic has led to a rapid shift towards digital health solutions, with telemedicine and online platforms becoming essential tools for healthcare services delivery. This accelerated transition redefined the critical role of eHealth literacy as a lifeline for health information navigation and utilization [6,7].

This competency is essential in the current era of open information where individuals’ health decisions and outcomes depend on their ability to discern trustworthy from unreliable sources of information [8]. Research indicates that individuals with high eHealth literacy levels are better equipped to navigate the sea of electronic health information, as they are able to critically evaluate the reliability and relevance of electronic health resources [9,10,11]. For instance, findings from a recent study highlight that those with advanced eHealth literacy skills are better equipped to differentiate between credible and non-credible health information, leading to a positive correlation with health behaviors and outcomes [10]. In contrast, a populace exhibiting lower eHealth literacy is inclined towards misinformation, with much higher discordance when it comes to confidence in conventional health information sources [12].

However, this skill does not always have positive effects. Cyberchondria is an illness anxiety-related disorder where the individual exhibits excessive online searching behavior for health related information [13]. Their online health information-seeking behavior exacerbates their anxiety and leads to distrust in healthcare providers [14]. A prior study in Saudi Arabia indicated that a high prevalence of “cyberchondria” was correlated with a high eHealth literacy level [15]. Another study also found that eHealth literacy predicted severe cyberchondria [16]. Individuals’ reliance on electronic platforms for health information exposes them to pseudoscience and disinformation, which may undermine their trust in healthcare providers [17,18]. This phenomenon has the potential to erode confidence in healthcare professionals [19]. Online health information-seeking behavior may negatively affect the patient–doctor relationship and undermine physicians’ authority [18,20].

Although many studies have explored eHealth literacy in various global settings, including the Middle East, few have investigated how individuals in the Middle East region evaluate sources of health information [2,21,22,23,24]. Public trust in healthcare providers varies between countries due to cultural and systematic differences, and there is little evidence in the literature on how eHealth literacy is related to the perceived trust in health information sources, including physicians and other healthcare providers [9,25,26,27].

The current study aimed to assess eHealth literacy levels along with the use and trust in various health information sources among the public in the Tabuk region of Saudi Arabia

## 2. Materials and Methods

### 2.1. Study Design and Timing

To assess the eHealth literacy level, usage, and trust in health information sources, a cross-sectional study was conducted from 21 December 2024 to 4 January 2025 among the public of the Tabuk region of Saudi Arabia.

### 2.2. Study Participants and Sampling

The participants in this study were individuals from the Tabuk region of Saudi Arabia who had access to the internet. The inclusion criteria were adults who were willing to participate. Individuals who work or study in the health field were excluded to avoid potential bias in their responses. This study utilized the snowball sampling technique to leverage social networks for rapid data collection.

### 2.3. Sampling Frame and Recruitment

To ensure the diversity of the sample and its representativeness of Tabuk populations, 15 social influencers in the Tabuk region, selected from different local social groups, were approached to distribute the survey link to their local network of contacts and community groups. They were teachers, lawyers, university students, engineers, accountants, sales managers, and soldiers. They worked either in private or public sectors in the Tabuk region, and two of them were retired. To ensure further diversity, the community respondents were encouraged to share the link with adult members of their family, relatives, and friends.

### 2.4. Sample Size Calculation

The current population of the Tabuk region of Saudi Arabia is 886,036, and nearly 99% have internet access [3,28]. The sample size was calculated using an online calculator, OpenEpi V3, which estimated a minimum sample size of 384 is required with a 95% confidence level and a 5% margin of error. The responses from 407 participants in this study were deemed sufficient for statistical analysis.

### 2.5. Data Collection Tool

Data were gathered through a self-administered online questionnaire hosted in Google Forms. The questionnaire was administered in Arabic and was designed to measure eHealth literacy, use, and trust in medical information sources, in addition to collecting demographic variables. The first page of the survey contained a consent form for participation in this study. The second page contained a question regarding the respondents’ background in health sciences. Respondents who either declined consent or replied that they have a health sciences background were excluded from continuing the survey. An online survey method was chosen to minimize social desirability bias, especially in rating trust level, which may occur during face-to-face surveys.

The questionnaire was initially pretested with 15 subjects to ensure clarity, readability, and feasibility. Based on the findings and the feedback from participants in this pilot study, the questionnaire was modified, refined, and finalized.

#### 2.5.1. Demographic Variables

The questionnaire included questions regarding age, gender, education, work, chronic disease status, and current medication use.

#### 2.5.2. Measuring eHealth Literacy

An Arabic-validated version of the eHealth literacy scale (eHEALS) was used to assess eHealth literacy skill [29]. Developed by Norman and Skinner (2006), this 8-item scale measures individuals’ perceived confidence and skills in finding, evaluating, and utilizing health-related information from electronic sources [4]. The participants rate their responses on a 5-point Likert scale for each item. The total score of eHEALS ranges from 8 to 40, with higher scores indicating a higher eHealth literacy level.

Two items were included in the questionnaire to measure the perceived importance and usefulness of internet health information. These items were not a part of the eHealth literacy scale calculation but intended to clarify the respondents’ general level of interest in electronic health information [4].

#### 2.5.3. Identifying the Main Source Used for Health Information

Guided by prior research findings and to make responses more informative and easier to interpret, the sources of health information were classified into five main categories: physicians and healthcare workers, family and friends, specialized health websites (e.g., Mayo Clinic, Altibbi, Webtib, etc.), other websites and search engines (not health-specific websites), and social media platforms (e.g., WhatsApp, Instagram, X, etc.) excluding accounts managed by official authorities or healthcare workers.

The participants were asked to select a single answer to the question “What is your main source of health information?” from the prior five categories of health information sources. This forced choice strategy aimed to replicate a decision-making process in the real world and to increase cognitive engagement for a more accurate result [30].

#### 2.5.4. Evaluating the Trust in Health Information Sources

For the five main categories of health information sources mentioned earlier, participants were asked to rate their trust into 5 levels (very untrustworthy, untrustworthy, neutral, trustworthy, and very trustworthy). These levels of trust were given scores, with a very untrustworthy score of 1, and very trustworthy received a score of 5.

### 2.6. Statistical Analysis

Statistical Package for Social Sciences (SPSS) Version 27 was used to analyze the survey data. Age, gender, education level, and employment position were among the sociodemographic characteristics for which descriptive statistics were computed. Student *t*-test and ANOVA were used to test the differences in eHealth literacy scale scores between dichotomous and nominal variables, while Spearman rank correlation was used to evaluate the association between eHealth literacy scale score and ordinal variables. Multiple linear regression analysis was used to assess the relationship between eHealth literacy and trust scores in each health information source, separately controlling demographic variables. A threshold of *p* < 0.05 was set to evaluate statistical significance, and all relevant *p* values were reported.

## 3. Results

**Figure 1:**
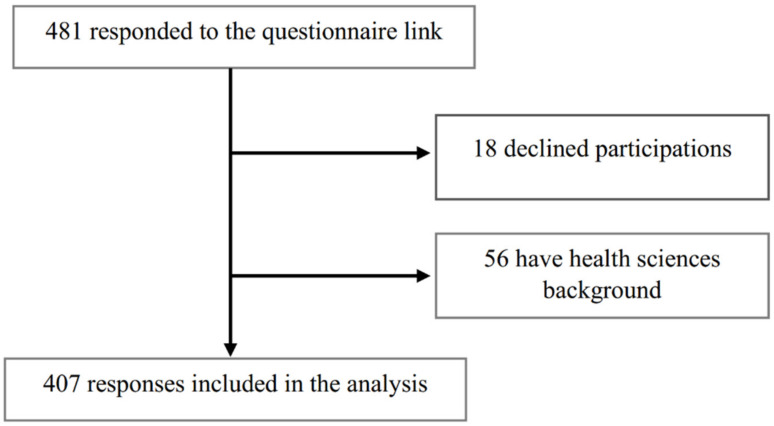
Flowchart of the selection process of study participants.

Among 481 who responded to the survey link, 407 responses were included in the final analysis (Figure 1). Table 1 below presents the demographic information of the study participants and their corresponding eHealth literacy scores. The respondents in this study attained a mean eHealth literacy score of 27.17 (SD = 5.98), and significant relationships between demographic variables and eHealth literacy were revealed. eHealth literacy scores decreased progressively with age (r = −0.254, *p* < 0.001), with participants over 65 years attaining the lowest scores (M = 20.00, SD = 6.93). Women demonstrated significantly higher eHealth literacy than men (*p* = 0.031). Education level was positively correlated with eHealth literacy (r = 0.234, *p* < 0.001), with higher education associated with higher scores. However, those with high school or below demonstrated a higher level of eHealth literacy compared to diploma holders. There was a statistically significant difference between work categories (F [4,402] = 5.51, *p* < 0.001), with non-working individuals having the highest mean score. Notably, respondents with chronic diseases (*p* = 0.012) and those currently on medications (*p* = 0.003) exhibited significantly higher eHealth literacy scores than their counterparts.

Table 2 presents participants’ responses regarding the perceived usefulness of the internet for health decision-making and the importance of accessing online health resources. A plurality of participants (43%) found the internet “useful” for making health decisions, while 32.2% were “unsure” about its utility. Only 6.6% considered it “very useful”, whereas 18.1% found it “not useful” or “not useful at all”. Asked about the importance of accessing health resources online, a similar pattern emerged, with 44% of the respondents stating that it was important and 32.4% being “unsure”. A small proportion (6.1%) considered it “very important”, while 17.4% viewed it as “not important” or “not important at all”.

Table 3 reveals the mean scores and standard deviations for the individual items on the eHEALS. The participants reported the highest levels of confidence when asked about distinguishing high-quality from low-quality health resources on the internet (M = 3.60, SD = 0.83) and using the internet to answer their health-related questions (M = 3.54, SD = 0.86). They also expressed confidence about using the health information that they found online (M = 3.54, SD = 0.92). The participants indicated slightly lower levels of knowledge about the health resources available online (M = 3.23, SD = 0.97), including where and how to find them (M = 3.29, SD = 1.02; M = 3.33, SD = 0.97).

Notably, the lowest mean score was observed for confidence about using online information to make health decisions (M = 2.96, SD = 1.01), suggesting that while the participants felt capable of finding and understanding online health information, they were more hesitant about applying this information to their health decision-making processes.

Table 4 below presents the list of five health information sources presented to the study participants, indicating the frequencies with which each was chosen as a respondent’s primary source. Most respondents (51.9%, *n* = 206) said that they relied on physicians and healthcare workers as their main source of health information. The second most common was “other websites and search engines” (18.1%, *n* = 72), followed closely by specialized health websites (15.4%, *n* = 61). The least-reported source was social media. This general preference for online sources (40%) reflects the growing trend in internet use for health information-seeking. In addition, family and friends were the primary sources of health information for 8.1% (*n* = 32) of the participants. These results suggest that, while digital sources are gaining popularity, healthcare professionals remain the most preferred source of health information for most people.

**Figure 2:**
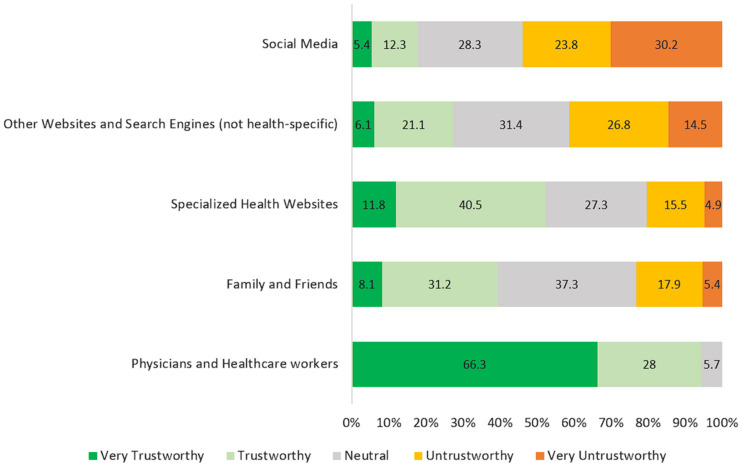
Participants’ trust in various sources of health information.

Figure 2 reveals the study participants’ levels of trust in various health information sources. Physicians and healthcare workers are overwhelmingly trusted, with 94.3% of participants rating them as either “Very trustworthy” (66.3%) or “Trustworthy” (28%). Only 5.7% remained neutral, and notably, no participants rated them “Untrustworthy”. Specialized health websites were the second most trusted source, with 52.3% of participants finding them trustworthy (11.8% “Very trustworthy” and 40.5% “Trustworthy”). However, 27.3% remained neutral, and 20.4% found them untrustworthy. Family and friends received mixed levels of trust: while 39.3% found them trustworthy, a larger portion (37.3%) remained neutral and 23.3% considered them untrustworthy. Other websites and search engines that do not specialize in health information were viewed with skepticism. Only 27.2% found them trustworthy, while 41.3% considered them untrustworthy and 31.4% remained neutral. Social media was the least trusted source, with 54% of participants rating it negatively (23.8% “Untrustworthy” and 30.2% “Very untrustworthy”). Only 17.7% found it trustworthy, while 28.3% remained neutral. These findings suggest that physicians and healthcare workers are the most trusted sources of health information, while social media and non-health-specialized websites are viewed with greater skepticism.

Table 5 presents a regression analysis examining the association between eHealth literacy and trust in various sources of health information, after controlling demographic factors. Higher eHealth literacy was associated with more trust in specialized health websites (B = 1.845, SE = 0.321, *p* < 0.001) and less trust in social media (B = −0.641, SE = 0.238, *p* = 0.007). There was a positive association between eHealth literacy and trust in physician healthcare workers (B = 0.864, SE = 460, *p* = 0.061). However, although the *p*-value approaches significance, it does not reach conventional levels (*p* < 0.05). Finally, there was no association between eHealth literacy and trust in family and friends and nonspecialized health websites.

## 4. Discussion

It is important to preface the discussion by emphasizing that the current study’s sample excluded individuals with medical knowledge or those employed in the medical field. This methodological decision was implemented to ensure that the responses represent the perspectives of the general public, uninfluenced by a background of medical knowledge.

The average eHealth literacy score of the respondents was 27.17, which is close to the results found with the results of other studies conducted in the Middle East [21,22,24]. A pre-pandemic study in Italy measured eHealth literacy specifically in the general public who do not have a health science background [31]. This study found eHealth literacy to be 26.7. Other studies in high-income countries like Singapore and Japan reported eHealth literacy scores of 23.44 and 23.4 respectively [32,33]. Interestingly, a study among university students in China found a steady increase in eHealth literacy levels during and after the pandemic period of COVID-19 [34]. In this study, the eHealth literacy score was 25.4 during the initial period of the pandemic, which peaked at 29.4 in 2023, reflecting the increased interest in digital health platforms [34]. This could explain the higher eHealth literacy scores in recent reports compared to the findings of older studies.

In this study, females showed higher eHealth literacy levels than males, consistent with the findings of prior studies [15,21,22,24]. In this study, the average eHealth literacy score among males and females was 26.58 and 27.86, respectively. Compared to the Jeddah study, the average score among males was 27.62, while the average score for females was 29.92. This slight difference may be due to the differing proportions of student participants in each study: 37.7% in the Jeddah study versus 11.3% in the current study. Generally, younger individuals tend to score higher on the eHealth literacy scale, which could explain the observed difference. Moreover, the higher literacy among females could be attributed to a greater interest in the topic of health and higher engagement in online health information seeking [35,36]. eHealth literacy levels progressively decreased with age, with the elderly showing the lowest levels. This finding could be explained by the lower level of digital competency among the elderly and the digital divide that they face [37]. Education level, as expected, was associated with a higher eHealth literacy level, corroborating the findings of prior research [6,21,22,24]. The finding in this study that the diploma holders have a lower level of eHealth literacy compared to those with high school degree can be attributed to several factors. Primarily, high school graduates tend to be younger and may possess more advanced technical skills. Additionally, many high school graduates are likely to be currently enrolled in university programs, indicating ongoing educational engagement. These factors collectively suggest that age, technological aptitude, and current educational status may be more significant determinants of eHealth literacy than formal educational attainment in this context. Furthermore, the finding that unemployed participants had a higher average level of literacy than public- and private-sector employees was in conflict with the findings of other studies [6,22,24]. This could be explained by the different work categories used in other studies, with work status potentially being an indirect measure of age or gender, as unemployment tends to be more prevalent among female and younger age groups. A quarter (26%) of the study participants had at least one chronic disease, and this group had a higher eHealth literacy level than those without a chronic disease. Those currently taking medication also had higher literacy levels. Prior research has produced mixed results in this area, with some indicating that individuals with chronic diseases struggle with electronic resources and others finding that chronic conditions are associated with higher eHealth literacy levels [22,38,39]. These differences can be explained by the digital divide between countries.

Physicians and healthcare workers are still considered the primary source of health-related information by the public. Alduraywish et al. (2020), in their study of visitors to a primary healthcare center, found that the majority saw physicians as their preferred source of health information. This was in line with the outcomes of a similar study conducted in the United States [2]. In Alduraywish et al.’s (2020) study, it was found that the second most commonly used source of health information was internet search engines, and the least commonly selected was social media. These findings are echoed by the current study, despite differences in the respective methodologies. Although this study used 5 options to reflect respondents’ trust level in various health information sources, none rated physicians and healthcare workers as untrustworthy. This is in line with the findings of Alduraywish et al. (2020), which show that no participants reported not trusting doctors [2]. The overall findings on perceived trust in different health information sources did not differ significantly from those of prior studies [2,35]. The highest level of trust was given to physicians and healthcare workers, followed by specialized health websites, family and friends, and general websites and search engines. Social media was the least trusted source.

A non-significant association was found between eHealth literacy and trust in physicians and healthcare providers after controlling sociodemographic factors. However, the *p* value was near the threshold (0.061). A recent study of 344 participants aged 44 years and older with a chronic disease found a positive association between eHealth literacy and trust in physicians, but that finding cannot be generalized to include healthy individuals, which the current study can [27]. Finally, the current study reveals that greater eHealth literacy was associated with more trust in specialized health websites and less trust in social media.

The present study is subject to the typical limitations inherent in a cross-sectional descriptive study that relies on a web-based self-administered questionnaire. The current study used a nonprobability sampling technique, which limits the generalizability of the findings. Response bias and the use of self-reported data may also have affected the accuracy of the responses. Further, the health information sources were not detailed for every possible platform or channel; instead, they were grouped into categories to decrease complexity and improve the clarity of the results. Other potential confounding factors—such as household income—were not included in this study, which may have impacted the analysis. However, it is worth mentioning that this is the first study to explore the relationship between eHealth literacy level and trust in health information sources in public with universal internet access.

## 5. Conclusions

This cross-sectional study examined eHealth literacy and trust in health information sources among the public in the Tabuk region of Saudi Arabia. This study suggested that despite the widespread availability of online health resources, physicians, and healthcare workers remained the primary and most trusted source of health information. Specialized health websites were moderately trusted, while social media platforms were the least trusted. Notably, higher eHealth literacy was significantly associated with increased trust in specialized health websites and distrust in social media. However, no significant relationship was found between eHealth literacy and trust in healthcare providers.

These results underscore the enduring preference for and trust in healthcare professionals as primary health information sources, even in populations with universal internet access in this era of open information. This trust was not associated with eHealth literacy level. The findings suggest that eHealth literacy empowers individuals to critically evaluate online content, fostering trust in credible platforms like specialized websites while discouraging reliance on less reliable sources such as social media.

Future work could evaluate eHealth literacy utilizing the more extensive eHealth Literacy Assessment Toolkit (eHLA) developed by the WHO, assess the trust in physicians using the Trust in Physician Scale (TiPS), and evaluate the interplay between these two factors. Future research should employ longitudinal designs to explore causal relationships between eHealth literacy and trust dynamics. Adopting probability sampling, and expanding demographic diversity, including rural populations and socioeconomic variables, could enhance generalizability. Additionally, interventions to improve eHealth literacy, particularly among older adults and less-educated groups, may further mitigate reliance on low-quality health information. This study contributes to understanding how digital competencies intersect with trust in health information, offering insights for public health strategies aiming to optimize health communication in the digital age without fear of adversely affecting the patient–doctor relationship.

## Figures and Tables

**Table 1:** Study participants’ characteristics and eHealth literacy levels.

Demographics		*n* (%)	eHealth Literacy Mean (SD)	Test Statistics	*p* Value
		407	27.17 (5.98)		
Age	18–25	79 (19.4)	28.63 (5.99)	r = −0.254	<0.001
	26–35	99 (24.3)	28.39 (5.90)		
	36–45	115 (28.3)	27.57 (4.51)		
	46–55	77 (18.9)	26.01 (5.86)		
	56–65	34 (8.4)	22.11 (7.49)		
	>65	3 (0.7)	20.00 (6.93)		
Gender	Male	220 (54.1)	26.58 (5.99)	t = −2.160	0.031
	Female	187 (45.9)	27.86 (5.85)		
Education	High School or Below	68 (16.7)	25.18 (5.08)	r = 0.234	<0.001
	Diploma	39 (9.6)	22.82 (7.71)		
	Bachelor’s	244 (59.9)	28.06 (5.52)		
	Master’s, PhD	56 (13.8)	28.71 (5.71)		
Work	Public Sector	291 (71.5)	27.18 (5.97)	F (4,402) = 5.51	<0.001
	Private Sector	25 (6.1)	28.36 (6.59)		
	Student	46 (11.3)	28.34 (5.02)		
	Retired	27 (6.6)	22.56 (6.33)		
	Not Working	18 (4.4)	29.28 (3.43)		
Chronic Diseases Status	Yes	106 (26)	28.42 (6.22)	t = 2.529	0.012
	No	301 (74)	26.73 (5.84)		
Currently on Medications	Yes	138 (33.9)	28.37 (6.23)	t = 2.944	0.003
	No	269 (66.1)	26.55 (5.76)		

**Table 2:** Usefulness of the internet for accessing health resources and making health decisions (expressed in frequency and percentage).

	Not useful at all	Not useful	Unsure	Useful	Very useful
How useful do you find the internet when making decisions about your health?	36 (8.8%)	38 (9.3%)	131 (32.2%)	175 (43%)	27 (6.6%)
	Not important at all	Not important	Unsure	Important	Very important
How important is it for you to be able to access health resources on the internet?	40 (9.8%)	31 (7.6%)	132 (32.4%)	179 (44%)	25 (6.1)

**Table 3:** eHealth literacy scale responses (eHEALS).

		Mean	SD
1	I know what health resources are available on the internet.	3.23	0.97
2	I know where to find helpful health resources on the internet.	3.29	1.02
3	I know how to find helpful health resources on the internet.	3.33	0.97
4	I know how to use the internet to answer my questions about health.	3.54	0.86
5	I know how to use the health information I find on the internet to help me.	3.54	0.92
6	I have the skills I need to evaluate the health resources I find on the internet.	3.47	0.92
7	I can tell high-quality health resources from low-quality health resources on the internet.	3.60	0.83
8	I feel confident about using information from the internet to make health decisions.	2.96	1.01

**Table 4:** Participants’ responses to the question, “What is your main source of health information”?

Source	Frequency	Percentage
Physicians and healthcare workers	206	51.9%
Family and friends	32	8.1%
Specialized health websites (Mayo Clinic, altibbi, etc.)	61	15.4%
Other websites and search engines (not health-specific)	72	18.1%
Social media (WhatsApp, Instagram, X, etc.)	26	6.5%

**Table 5:** Regression analysis of health literacy and trust in different sources of health information.

Health Information Source	B	SE	*p* Value
Physicians and healthcare workers	0.864	0.460	0.061
Family and friends	0.065	0.281	0.818
Specialized health websites	1.845	0.321	<0.001
Other websites and search engines (not health-specific)	0.291	0.055	0.241
Social media	−0.641	0.238	0.007

Adjusted for age, gender, education, work, and medical history. B = regression coefficient, SE = standard error.

## Data Availability

The data presented in this study are available on reasonable request from the corresponding author (due to ethical and legal constraints).
